# Genome sequence of *Coxiella burnetii* strain Namibia

**DOI:** 10.1186/1944-3277-9-22

**Published:** 2014-12-29

**Authors:** Mathias C Walter, Caroline Öhrman, Kerstin Myrtennäs, Andreas Sjödin, Mona Byström, Pär Larsson, Anna Macellaro, Mats Forsman, Dimitrios Frangoulidis

**Affiliations:** 1 Institute of Bioinformatics and Systems Biology, Helmholtz Zentrum München, German Research Center for Environmental Health, Neuherberg, Germany; 2 Department of Genome-Oriented Bioinformatics, Center of Life and Food Science Weihenstephan, Technische Universität München, Freising, Germany; 3 CBRN Defence and Security, Swedish Defence Research Agency (FOI), Umeå, Sweden; 4 Bundeswehr Institute of Microbiology (BwIM), Munich, Germany

**Keywords:** *Coxiella burnetii*, Q fever, Whole genome sequencing, Next generation sequencing (NGS), Assembly, Annotation, Whole genome amplification

## Abstract

We present the whole genome sequence and annotation of the *Coxiella burnetii* strain Namibia. This strain was isolated from an aborting goat in 1991 in Windhoek, Namibia. The plasmid type QpRS was confirmed in our work. Further genomic typing placed the strain into a unique genomic group. The genome sequence is 2,101,438 bp long and contains 1,979 protein-coding and 51 RNA genes, including one rRNA operon. To overcome the poor yield from cell culture systems, an additional DNA enrichment with whole genome amplification (WGA) methods was applied. We describe a bioinformatics pipeline for improved genome assembly including several filters with a special focus on WGA characteristics.

## Introduction

Creation of whole genome information of *Coxiella burnetii* is a cumbersome procedure. All work with living strains of *C. burnetii* is impaired by the necessity to handle strains under Biosafety 3 conditions. The enrichment of this bacterium is normally done in animal derived cell culture systems with a peak of replication after 5 to 7 days of growth. The overall yield of bacteria, however, is less than that obtained by “classical” growth of bacteria on artificial media. Alternative enrichment methods, like animal inoculation and cultivation in hen eggs, present various problems and risks in processing, thus are not in common use for *C. burnetii*. The required amount and quality of DNA for whole genome sequencing of *C. burnetii* is not easily obtained by cell culture. Furthermore, DNA isolation is not always successful and not all DNA preparations are of a quality suitable for sequencing purposes. To overcome these problems, WGA techniques may present an attractive alternative for generation of *C. burnetii* DNA [1]. Such assays possess an impressive power to amplify traces of DNA to a satisfactory quantity. However, a careful evaluation with the species of interest is mandatory to judge its suitability. Repeat structures and insertions sequences (IS) in particular might influence the quality of amplification. The *Coxiella* genome shows IS elements with sometimes more than 100 copies [2], stressing the importance of thorough evaluation of WGA techniques. We chose a special variant of WGA, the MDA method, that has been successful applied [3,4] and is commercially available from different companies (RepliG, Qiagen, Hilden, Germany and GenomiPhi, GE Healthcare, Freiburg, Germany) [5]. Very recently the RepliG kit was used with *Coxiella* DNA and evaluated at 20 selected loci [6].

In this study, we describe a method for obtaining high quality DNA from *Coxiella* suitable for whole genome sequencing. We also evaluate the utility of WGA for *Coxiella* whole genome sequencing and WGA induced demands on downstream bioinformatics processing of sequence data, especially for genome assembly and finishing. The whole genome sequence presented here is the first of a *C. burnetii* strain originating from the African continent and will increase the genomic knowledge for this region.

## Organism information

*C. burnetii* is the causative pathogen of the zoonotic disease Q fever, which has a worldwide distribution with the only exceptions of New Zealand and Antarctica. The bacterium was first independently described and isolated in Australia and the United States of America in 1937 [7,8]. *C. burnetii* is an obligately intracellular, small, Gram-negative, non-motile, pleomorphic, coccobacillary bacterium (0.2 – 0.4 μm × 0.4 – 1 μm). Atypically, its Gram-negative membrane cannot be stained using Gram techniques, but can be visualized by the Gimenez method [9].

As a result of phenotypic similarities, the genus *Coxiella* was initially placed within the *Rickettsiales* order. More recent phylogenetic investigations, mainly based on 16S rRNA gene sequence analysis, resulted in re-classification of the *Coxiella* genus into the *Legionellales* order [10]. Within the *Proteobacteria*, they belong to the family *Coxiellaceae*[11] (Table 1).

**Table 1:** **Classification and general features of *C. burnetii *** **strain Namibia according to the MIGS recommendations**[33]

**MIGS ID**	**Property**	**Term**	**Evidence code**^**a**^
	Classification	Domain: *Bacteria*	TAS [34]
		Phylum: *Proteobacteria*	TAS [35]
		Class: *Gammaproteobacteria*	TAS [36,37]
		Order: *Legionellales*	TAS [10,38]
		Family: *Coxiellaceae*	TAS [11]
		Genus: *Coxiella*	TAS [39]
		Species: *Coxiella burnetii*	TAS [7,8,40]
		Strain: Namibia	NAS
	Gram stain	Negative	TAS [14]
	Cell shape	Coccobacillary rod	TAS [14]
	Motility	None	TAS [14]
	Sporulation	No*	TAS [12]
	Temperature range	35 – 37°C	TAS [26]
	Optimum temperature	37°C	TAS [26]
	pH range; Optimum	4.5-5.3; 4.5	TAS [41]
	Carbon source	Glutamate, citrate	TAS [42,43]
MIGS-6	Habitat	intracellular, polyhostal long persistence in the environment	TAS [26]
MIGS-6.3	Salinity	Unknown	NAS
MIGS-22	Oxygen	Microaerophilic (2.5%)	TAS [26]
MIGS-15	Biotic relationship	Endosymbiont	NAS
MIGS-14	Pathogenicity	highly pathogenic	TAS [44]
MIGS-4	Geographic location	Windhoek, Namibia	NAS
MIGS-5	Sample collection	1991	NAS
MIGS-4.1	Latitude	Unknown	NAS
MIGS-4.2	Longitude	Unknown	NAS
MIGS-4.4	Altitude	Unknown	NAS

^a^Evidence codes - IDA: Inferred from Direct Assay; TAS: Traceable Author Statement (i.e., a direct report exists in the literature); NAS: Non-traceable Author Statement (i.e., not directly observed for the living, isolated sample, but based on a generally accepted property for the species, or anecdotal evidence). These evidence codes are from the Gene Ontology project [45].

*A morphological distinct variant with enhanced stability in harsh environmental conditions is described (SCV = small cell variant).

In its development cycle, *C. burnetii* generates both large (LCV) and small cell variants (SCV). The latter are more environmentally stable and present the infectious particles incorporated by different hosts. After uptake by macrophages, the LCV is formed within phagolysosomes.

The bacterium exists in two antigenic phases, which are analogous to the smooth (phase I) and rough (phase II) LPS forms seen among the *Enterobacteriaceae.* Phase I bacteria can be observed during natural infections of humans and animals, whereas bacteria in phase II, which are mainly non-virulent, evolve after several passages in embryonated hen eggs or cell cultures. Transitions between both forms have been described [12].

*C. burnetii* has a large reservoir of hosts including many wild and domestic mammals, birds, reptiles, fish and even arthropods such as ticks and flies. Due to its transmission by inhalation, low infectious dose, high stability, and prior weaponization, *C. burnetii* is classified as a category B agent of bioterrorism by the Centers for Disease Control (CDC, Atlanta, USA) [11]. Epidemiological studies have demonstrated that the most frequent route of human *C. burnetii* infections is via domestic ruminants such as sheep, goats, or cattle. These animals may be chronically infected without showing any clinical symptoms and shed vast numbers of the bacterium into the environment, mainly during parturition. Counts of *C. burnetii* in excess of 10^9^ bacteria per gram have been recorded in placental tissue, but in other birth-associated products such as amniotic fluids or in milk, high quantities of *C. burnetii* may also be present. Particularly high counts have been obtained from tick feces with reports of 10^10^ living organisms per gram [13]. Despite this, ticks do not appear to be a significant risk factor for acquisition of human infection [14].

The organism is a highly infectious agent, with experimental estimates suggesting an infectious dose of less than 10 organisms for manifestation of an infection [15]. Furthermore, coxiellae are highly resistant to both heat and desiccation, ubiquitously available, and their aerosolized state is infectious over several kilometers [16,17].

*C. burnetii* strains appear with five different plasmid types, four different plasmids (QpH1, QpRS, QpDV, and QpDG) and one type with a chromosomal plasmid-homologous sequence [18-22]. The characterization of these plasmids led to a classification into five genomic groups. Some plasmid types could be associated with various geographic regions. A formerly hypothesized correlation of these genomic groups with virulence or clinical manifestation could not be confirmed in later studies [23].

**Figure 1:**
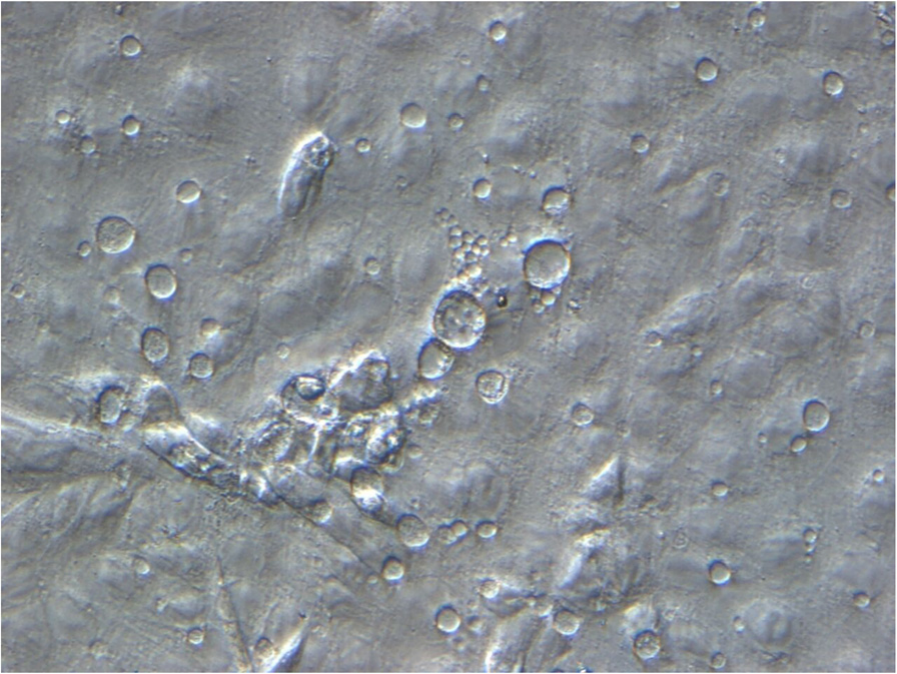
***C. burnetii-*** **infected BGM cells displaying the typical intracellular vacuoles (400× Hoffman modulation contrast image; E. Schröpfer and D. Frangoulidis).**

**Figure 2:**
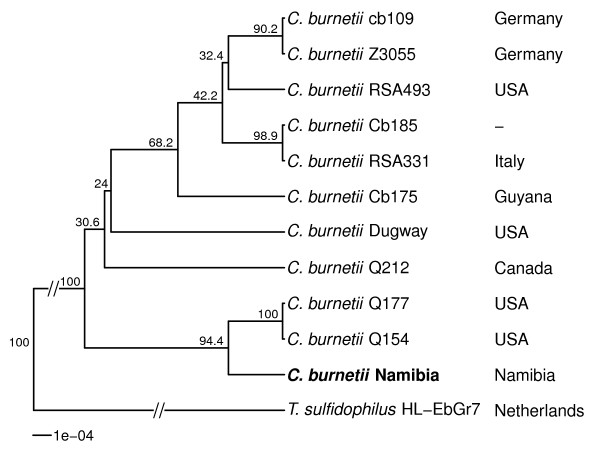
**Phylogenetic tree highlighting the position of *C. burnetii *strain Namibia (shown in bold) relative to the other *C. burnetii *strains with whole genome sequences available.** The average linkage (UPGMA) tree was inferred from 5,010 aligned positions of conserved blocks (determined using Gblocks [27]) of the rRNA operon sequences using the Juxes & Cantor model, calculated with the R packages ape [28] and phangorn [29]. Bootstrap values (expressed as percentages of 1,000 replicates) are shown at branch points. The closest related species based on a BLAST [30] search against bacterial genomes of the National Center for Biotechnology Information (NCBI) non-redundant (nr) database [31] using the rRNA operon sequence is currently *Thioalkalivibrio sulfidophilus* HL-EbGr7 [32], a species commonly isolated from soda lakes, and was used as outgroup to root the tree.

Because of the highly infectious nature of *C. burnetii,* cultivation should not be attempted outside adequate BSL 3 laboratories. Even with these, isolation is a difficult and very time-consuming procedure. Moreover, culture is not as sensitive as other methods such as detection of *Coxiella*-specific DNA. Viable cultures are however necessary for further scientific investigations, thus remain a research if not diagnostic priority. As *C. burnetii* is a strict intracellular bacterium, options for cultivation were previously restricted to the use of guinea pigs, mice and embryonated eggs [24]. These have now been largely abandoned for safety reasons. Instead, the less hazardous *in vitro* use of cell cultures such as human embryonic lung fibroblasts (HEL cells); embryonic epithelial kidney cells like the BGM cells; Vero-Cells or L929 have become the mainstay for cultivation work [25]. The culture of *C. burnetii* may require several weeks prior to the appearance of intracellular vacuoles, the hallmark of successful infection (see Figure 1). Although *C. burnetii* is an obligate intracellular pathogen, that needs living cells for cultivation, Omsland et al. recently published the development of a complex nutrient medium that supported substantial growth of *C. burnetii* in a 2.5% oxygen environment under axenic (host cell-free) conditions [26].The strain Namibia was first described in 1991, when isolated from an aborting goat. It shows the QpRS plasmid type, which is rarely observed, compared to the predominant QpH1 variant. Using established molecular typing methods, the strain shows the sequence type (ST) 30 (Multispacer Sequence Typing = MST) and the Multiple Loci Variable number of tandem repeat (VNTR) analysis (MLVA) genotype D16. Based on these typing data, the nearest known geographic neighbor is a strain from Morocco, which belongs also to the D cluster (D6). However, the Morocco strain has a very different repeat pattern and currently no whole genome sequencing data is available to determine its phylogenetic relationship. The phylogenetic position of strain Namibia is show in Figure 2.

## Genome sequencing information

### Genome project history

The organism was initially selected for the development of a rapid Melt-MAMA SNP typing [46] on the basis of its economic importance in livestock farming and public health as well as its geographical location. It is the first known sequenced isolate from the African continent having the QpRS plasmid. The genome was sequenced in 2012 and the pre-filtered sequencing data was deposited at the NCBI Short Read Archive (SRA) under the accession SRX270891 and made public in September 2013. The assembly and annotation version was deposited at DDBJ/EMBL/GenBank with the accession numbers CP007555 and CP007556 and locus tag CBNA. Table 2 presents the project information and its association with MIGS version 2.0 compliance [33].

**Table 2:** Project information

**MIGS ID**	**Property**	**Term**
MIGS-31	Finishing quality	Improved high-quality draft
MIGS-28	Libraries used	Nextera DNA Sample Prep Kit
MIGS-29	Sequencing platforms	Illumina MiSeq, 2x 150 paired-end
MIGS-31.2	Fold coverage	91x
MIGS-30	Assemblers	SPAdes, IDBA
MIGS-32	Gene calling method	Prodigal, GeneMarkS, Glimmer
	Locus Tag	CBNA
	NCBI Taxonomy ID	1321945
	Genbank ID	CP007555, CP007556
	Genbank Date of Release	October 16, 2014
	GOLD ID	Gi0055848
	BIOPROJECT	PRJNA197124
	Project relevance	Medical, bioforensic, evolution
MIGS-13	Source Material Identifier	SAMN02045684

### Growth conditions and DNA isolation

BGM cells (Flow Laboratories, Rockville, MD, USA) were grown in Eagles minimal essential medium (MEM) supplemented with Earls salts, 2 mM L-glutamax, 5% Fetal Calf Serum (FCS), 1% Non-Essential Amino acids (NEA) and 0.2% sodium bicarbonate (Sigma-Aldrich, St Louis, MO, USA). Confluent cell layers were infected with bacteria and incubated at 37°C. Fresh media was added after 20–24 h. To enhance vacuole formation, the infected confluent BGM cells were divided using trypsin. *C. burnetii* cells were collected from the medium of actively growing cultures after 7–8 days by differential centrifugation. An initial centrifugation step to remove cell debris was performed at 500 × g (1,500 rpm) for 5 minutes at 4°C, followed by a second centrifugation step to collect the bacteria at 2,550 × g (3,500 rpm) for one hour at 4°C.

The bacteria from confluently growing cell cultures were harvested by differential centrifugation as described above. The bacteria were washed twice in PBS. The bacterial pellet was then resuspended in 50 mM Tris (pH 7.8) and mixed with 10 mM MgSO_4_ solution containing 20 μg DNase (Ambion, Life Technologies, Carlsbad, CA, USA). The resulting suspension was incubated at 37°C for 30 minutes. 0.5% SDS and 50 μg/ml proteinase K solution were then added and the sample was incubated at 56°C for one hour. After cooling to room temperature, 100 mM Tris (pH 7.8), 1 mM EDTA, a 15% sucrose solution, and 1 mg/ml lysozyme solution (Sigma-Aldrich) were added and the resulting mixture was incubated at 37°C for 16 h. On the following day, 100 mM Tris (pH 12.0), 1 mM EDTA, and 5% SDS were added and the sample was incubated at 56°C for one hour.

The sample was then cooled to room temperature and treated with phenol/chloroform twice before three volumes of ice cold (−20°C) 99.5% ethanol to precipitate the DNA were added. After incubation at −20°C for 30 min, the sample was centrifuged at 19,000 × g (15,000 rpm) for 30 min. The pellet was then resuspended in 1 × TE containing 50 μg RNase (Epicentre, Madison, WI, USA) and incubated at 37°C for one hour. Proteinase K (500 μg, Epicentre) was then added and the resulting mixture was incubated for another hour at 37°C. The sample was treated with phenol/chloroform twice before precipitation of the DNA by adding 0.1 volume of 3 M sodium acetate and 2.5 volumes of ice cold (−20°C) 99.5% ethanol. The resulting mixture was then incubated at −20°C for 30 min, centrifuged, and washed twice with 80% ethanol. After centrifugation the pellet was air dried and resuspended in 1 × TE.

To obtain larger quantities of DNA for whole genome sequencing, the sample was amplified using the MDA kit Illustra GenomePhi V2 Amplification Kit (GE Healthcare Life Sciences and the REPLI-g UltraFast Mini Kit (Qiagen, Hilden, Germany), respectively, according to the manufacturers’ instructions. Once the amplification was complete, the enzymes were inactivated by heating the sample to 65°C. The product was then diluted with sterile distilled water, the DNA was extracted using phenol/chloroform, and the product was precipitated using 0.1 volumes of 3 M sodium acetate and 2.5 volumes of ice cold (−20°C) 99.5% ethanol followed by incubation for 16 h at −20°C. The precipitated sample was centrifuged at 19,000 × g (15,000 rpm) for 30 minutes at 4°C, washed once with 70% ethanol and air dried. The pellet was then dissolved in 1 × TE and the DNA concentration was estimated using a Qubit fluorometer (Life Technologies, Carlsbad, CA, USA).

### Genome sequencing and assembly

The isolated DNA was prepared using the Nextera DNA Sample Prep Kit (Illumina, Hayward, CA, USA) and paired-end reads of 150 bp were sequenced on a MiSeq benchtop sequencer (Illumina) at the Swedish Defence Research Agency and according to the manufacturer’s instructions.

The sequenced reads were filtered against the draft genome assembly of *Chlorocebus sabaeus* (assembly AQIB01), *Macaca mulatta* (assembly AANU01), and *Papio anubis* (assembly AHZZ01) as well as four bacterial contaminants: *Escherichia coli* str. K-12 substr. MG1655 (NC_000913), *Mycoplasma arginini* 7264 (NZ_AORG01000000), *Propionibacterium acnes* 6609 (NC_017535) and *Streptococcus suis* SC84 (NC_012924) using mirabait, a kmer-based read mapping tool [47]. Afterwards, bacterial contaminant reads were blasted against the *C. burnetii* RefSeq genomes and the contaminant genomes. Reads which are more similar in their full length to *C. burnetii* were reintegrated into the filtered read set. This step was done as a quality control not to filter too many reads, such as reads mapping to orthologous genes or the ribosomal RNA operon.

Afterwards we used MIRA [47] to quality trim the reads and aligned them to the closest strain Q154 [48]. The raw coverage at this stage was 120×. Then, we used BayesHammer [49] to correct the Illumina reads followed by COPE [50] to merge overlapping reads (about 18%). The resulting merged and unmerged paired-end reads were assembled using Velvet-SC [51], SPAdes [52] and IDBA-UD [53], three assemblers optimized for single-cell sequence data with unequal coverage. Finally, we used GAM-NGS [54] to merge the contigs of the resulting assemblies. During the whole assembly process we used QUAST [55] to find the optimal parameters to obtain as few contigs as possible with the least number of misassemblies and InDels and the greatest N50 value. Afterwards, we used Mauve [56] to predict the order and orientation of the contigs corresponding to Q154.

A close inspection of the contig boundaries revealed many a lot of chimeric sequences, especially at nearly all of the possible IS1111 insertion sequence [57] sites. The reason for this frequent chimera formation can be explained by circular intermediates of IS1111 (although their presence in Nine Mile could not be detected by PCR, maybe caused by low expression level of these transposases) in combination with the whole genome amplification technique applied here [58,59].

We used Cutadapt [60] to trim IS1111 sequences from the 5’ and 3’ end of the assembled contigs to avoid mis-scaffolding and artificial gap filling. Afterwards, we used Opera [61] and information from our extended IS1111 typing method (manuscript in preparation) to scaffold the contigs semi-automatically. Then we used GapFiller [62] to close or reduce gaps. All insertion sequences sites (including IS1111, IS30 and ISAs1) were verified again to avoid false positive insertions and to fill in missing sequences with Ns.

### Genome annotation

Gene calling and functional annotation was performed using the PEDANT system [63]. Briefly, genes were called using Prodigal [64], Glimmer [65] and GeneMarkS [66]; all had been trained on the five RefSeq complete *C. burnetii* genomes. Consensus gene models were created by majority, domain or structural annotations in alternative start regions or preferring the Prodigal model. Structural RNAs were predicted using RNAmmer [67] (rRNAs), tRNAscan-SE [68] and similarity to Rfam [69]. The known 23S rRNA intervening sequence (IVS) [70] and the two self-splicing group I introns [71] were annotated manually. Protein similarities and InterPro domain annotations were obtained from SIMAP [72] if possible or computed locally. Similarities to SCOP [73] and KEGG [74] were computed using BLAST [30]. Signal peptides were predicted using SignalP [75], transmembrane proteins using TMHMM [76]. Gene names and protein descriptions were annotated by a combination of a stringent similarity search against the UniProtKB/Swiss-Prot database [77] as well as using BLANNOTATOR [78] followed by manual curation. The genome and its functional annotation can be browsed at the PEDANT website [79].

## Genome properties

**Figure 3:**
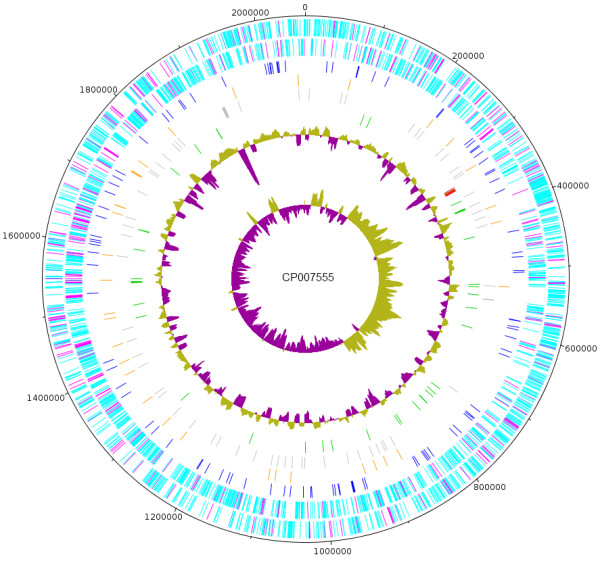
**Graphical circular map of the chromosome.** From outside to the center: Genes on forward strand (color by ‘with function prediction’ turquoise or hypothetical magenta), Genes on reverse strand (color scheme is the same as on forward strand), pseudogenes (blue), insertion elements (orange), gaps (gray), RNA genes (tRNAs green, rRNAs red), GC content, GC skew.

**Figure 4:**
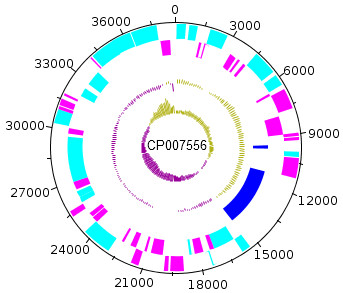
**Graphical circular map of the plasmid.** From outside to the center: Genes on forward strand (color by ‘with function prediction’ turquoise or hypothetical magenta), Genes on reverse strand (color scheme is the same as on forward strand), pseudogenes (blue), GC content, GC skew.

The *C. burnetii* Namibia genome has a total size of about 2,101,438 bp (41.1% GC content), with one circular chromosome of about 2,062,778 bp (containing 61 gaps with an estimated total gap length of 66,246 bp) and one plasmid of 38,660 bp. For the chromosome and plasmid, 2,030 genes were predicted, 1,979 of which are protein-coding genes. 1,309 of protein coding genes were assigned to a putative function with the remaining annotated as hypothetical proteins. 21 protein coding genes belong to 9 paralogous families in this genome corresponding to a gene content redundancy of 0.9%, mainly caused by the high number of transposases. The properties and the statistics of the genome are summarized in Tables 3, 4 and 5 and a circular map of the chromosome and plasmid is shown in Figures 3 and 4.

**Table 3:** Summary of genome: one chromosome and one plasmid

**Label**	**Size (Mb)**	**Topology**	**INSDC identifier**
Chromosome	2.06	circular	CP007555
Plasmid	0.04	circular	CP007556

**Table 4:** Genome statistics

**Attribute**	**Value**	**% of Total**^**a**^
Genome size (bp)	2,101,438	100.00
DNA coding (bp)	1,788,283	82.88
DNA G + C (bp)	865,056	41.16
DNA scaffolds	2	100.00
Total genes	2,030	100.00
Protein coding genes	1,979	97.49
RNA genes	51	2.51
Pseudo genes	98	4.83
Genes in internal clusters	21	1.03
Genes with function prediction	1,309	66.14
Genes assigned to COGs	1,294	65.39
Genes with Pfam domains	1,424	71.96
Genes with signal peptides	263	13.29
Genes with transmembrane helices	473	23.90
CRISPR repeats	NA	

^a^The total is based on either the size of the genome in base pairs or the total number of protein coding genes in the annotated genome.

**Table 5:** Number of genes associated with general COG functional categories

**Code**	**Value**	**% age**	**Description**
J	135	6.82	Translation, ribosomal structure and biogenesis
A	1	0.05	RNA processing and modification
K	50	2.53	Transcription
L	90	4.55	Replication, recombination and repair
B	0	0.00	Chromatin structure and dynamics
D	28	1.41	Cell cycle control, Cell division, chromosome partitioning
V	23	1.16	Defense mechanisms
T	40	2.02	Signal transduction mechanisms
M	120	6.06	Cell wall/membrane biogenesis
N	12	0.61	Cell motility
U	37	1.87	Intracellular trafficking and secretion
O	59	2.98	Posttranslational modification, protein turnover, chaperones
C	89	4.50	Energy production and conversion
G	74	3.74	Carbohydrate transport and metabolism
E	105	5.31	Amino acid transport and metabolism
F	46	2.32	Nucleotide transport and metabolism
H	93	4.70	Coenzyme transport and metabolism
I	62	3.13	Lipid transport and metabolism
P	47	2.37	Inorganic ion transport and metabolism
Q	32	1.62	Secondary metabolites biosynthesis, transport and catabolism
R	155	7.83	General function prediction only
S	104	5.26	Function unknown
-	685	34.61	Not in COGs

The total is based on the total number of protein coding genes in the genome.

## Insights from the genome sequence

A whole genome comparison with all five complete reference genomes (accessions: NC_009727, NC_010117, NC_002971, NC_011528, NC_011527) revealed strain Q154 as the most similar strain. In silico typing of strain Namibia showed the same *adaA* deletion variant 1 [80], but Multispacer Sequence Typing (MST) and Multiple-locus variable-number of tandem repeat (VNTR) analysis (MLVA) generated different profiles compared to Q154 (30 vs. 8 and D16 vs. D6 respectively) [46,81].The COG distribution is quite similar, except fewer annotated proteins in Q154, likely because of older gene prediction algorithms. The tRNA composition is identical in both strains. At the nucleotide level 2,767 chromosomal SNPs and 77 plasmid-related SNPs were found (752 intergenic, 5 non-coding and 2,087 within coding regions). Further, there is a 6 kb region in the Namibia genome which is not present in the Q154/Q177 clade (Figure 2) but in the other complete reference strains. It contains the ankyrin repeat protein AnkI (CBNA_1063). Also, a 4.5 kb region present in Q154 (containing an acetyltransferase, CbuK_0095 and a bacterial regulatory protein, CbuK_0101) is absent in Namibia. Large structural variations were not detected.

## Conclusion

We present the first whole genome sequence of *Coxiella burnetii* strain Namibia from Africa with its distinct genotype and unique genomic features and regions. We describe a combined set of laboratory methods and bioinformatics tools that resulted in a high quality whole genome sequence of this strain. The applied bioinformatics approach accounts for potential problems caused by the MDA/WGA method such as uneven sequence coverage and artificial products like chimeric reads. The sequencing and assembly pipeline presented here is suggested as a standard when sequencing of *C. burnetii* strains is done with or without the application of whole genome amplification methods. The incorporation of insertion sequence typing data can help to reduce the number of scaffolds down to a single whole genome sequence and avoids creating and sequencing an additional long distance mate-pair library usually needed to scaffold highly repetitive genomes.

To speed up the sequencing of new *C. burnetii* strains and to overcome the problems in generating high quality genomes, a joint research project with the Swedish Defence Institute (FOI) in Umeå was established: The Coxiella Genome Sequencing Consortium (CGSC) [82].

## Abbreviations

WGA: Whole genome amplification; MDA: Multiple displacement amplification; BGM: Buffalo Green Monkey.

## Competing interests

The authors declare that they have no competing interests.

## Authors’ contributions

MB, AM performed the microbiology and molecular biology studies; CÖ, KM performed the sequencing; MCW performed the annotation and genomic analysis; DF provided strain material; AS, PL, MF, DF, MCW wrote the manuscript. All authors read and approved the final manuscript.
